# Paraffin sections may be useful in Mohs micrographic surgery for nonmelanoma skin cancer in select patients

**DOI:** 10.1016/j.jdin.2024.07.005

**Published:** 2024-08-02

**Authors:** Ryan T. Ladd, Taylor Viggiano, Sarah E.B. Smith, Skye Buckner-Petty, Yul W. Yang, Shari A. Ochoa

**Affiliations:** a Mayo Clinic Arizona Department of Dermatology, Scottsdale, Arizona; b Mayo Clinic Arizona Division of Clinical Trials and Biostatistics, Scottsdale, Arizona

**Keywords:** change in management, Mohs micrographic surgery, nonmelanoma skin cancer, paraffin sections, permanent sections, upstaging

*To the Editor:* Sending paraffin sections during Mohs micrographic surgery (MMS) is a longstanding practice and standard for melanocytic lesions; however, the indications for paraffin sections for nonmelanoma skin cancer (NMSC) are less clear.^1^, ^2^, ^3^ Here, we review the utility of sending paraffin sections during Mohs surgery for NMSC.

A single-center retrospective review was performed between 2009 and 2020 on patients who underwent MMS for NMSC with the use of paraffin-embedded sections at the Mayo Clinic in Scottsdale, Arizona. Surgeries were performed by a fellowship-trained Mohs surgeon who had paraffin sections sent for select high-risk NMSC cases. Patient records were reviewed for demographics, tumor characteristics, and indications for sending paraffin sections—further characterized as unexpected histology, upstaging, or to ensure clearance.

From 2009 to 2020, a total of 11,083 NMSC sites were treated with MMS, with 127 tumors (0.24%) identified and submitted for debulk analysis. Notably, 22 individuals (19%) had a history of organ transplant. Tumor location, histologic subtype, and recurrence rate were recorded and reported in Table I. The most common reason for paraffin sections was to assess for upstaging characteristics (69%), particularly for squamous cell carcinoma (SCC). Notably, all patients who had received an organ transplant within our cohort (22 patients) had paraffin sections sent for SCC, accounting for nearly one-third of the SCC patients. Overall, paraffin sections changed management in 25% of cases, predominantly in those with SCC (30% of cases). Radiation and additional surgery were the most common interventions offered as changes in management.

**Table I:** Characteristics of tumors (*N* = 127) treated with Mohs surgery when paraffin sections were utilized

	BCC	SCC	Other
Tumor type (%)	32/127 (25.2%)	86/127 (67.7%)	9/127 (7.1%)
Location	**Head/neck:** 31/32 (96.9%)	**Head/neck:** 79/86 (91.9%)	**Head/neck:** 4/9 (44.4%)
	**Trunk:** 1/32 (3.1%)	**Trunk:** 2/86 (2.3%)	**Trunk:** 3/9 (33.3%)
	**Extremities:** 0	**Extremities:** 5/86 (5.8%)	**Extremities:** 2/9 (22.2%)
Subtypes	**Superficial:** 0/32 (0%)	**SCCIS:** 2/86 (2.3%)	**DSFP:** 5/9 (55.6%)
	**Nodular:** 12/32 (37.5%)	**Well diffˆ:** 22∗/86 (25.6%)	**AFX:** 1/9 (11.1%)
	**Metatypical:** 1/32 (3.1%)	**Moderately diff:** 34/86 (39.5%)	**Basaloid epithelial tumor:** 1/9 (11.1%)
	**Infiltrative/micronodular:** 19/32 (59.3%)	**Poorly diffˆ:** 28∗/86 (32.6%)	**Microcystic adnexal carcinoma:** 1/9 (11.1%)
			**Trichilemmal carcinoma:** 1/9 (11.1%)
Recurrent tumor	**Yes:** 3/32 (9.4%)	**Yes:** 5/86 (5.8%)	**Yes:** 0
	**No:** 29/32 (90.6%)	**No:** 81/86 (94.2%)	**No:** 9/9 (100%)
Reason for paraffin sections	**Ensure Clearance:** 2/32 (6.3%)	**Ensure Clearance:** 2/86 (2.3%)	**Ensure Clearance:** 6/9 (66.7%)
	**Unexpected Histology:** 19/32 (59.4%)	**Unexpected Histology:** 9/86 (10.5%)	**Unexpected Histology:** 1/9 (11.1%)
	**Concern for Upstaging:** 11/32 (34.3%)	**Concern for Upstaging:** 75/86 (87.2%)	**Concern for Upstaging:** 2/9 (22.2%)
Change in management	**Yes (total):** 4/32 (12.5%)	**Yes (total):** 26/86 (30.2%)	
	**Radiation:** 4/32” (12.5%)	**Radiation:** 23/86 (26.7%)	
	**Additional surgery:** 2/32” (6.3%)	**Additional surgery:** 3/86 (3.5%)	

ˆ, well differentiated ≈ low grade; poorly differentiated ≈ high grade; ∗, lack of clear comment on differentiation in pathology report; ”, change of management resulted in both surgery and radiation.

*AFX*, Atypical fibroxanthoma; *BCC*, basal cell carcinoma; *diff*, differentiation; *DSFP*, dermatofibrosarcoma protuberans; *Ensure Clearance*, ensure tumor clearance; *SCC*, squamous cell carcinoma; *SCCIS*, squamous cell carcinoma in situ; *Upstaging*, further evaluate lesions in staging work-up or for perineural or perivascular invasion.

Our study demonstrates the utility of paraffin sections in MMS for high-risk NMSC cases, particularly SCC, where permanent sections frequently prompted management changes, especially in immunocompromised patients. Our observed upstaging rate of 30% for SCC aligns with prior studies by Nemeh et al, highlighting the added diagnostic value of debulk analysis not initially apparent in MMS frozen sections alone.^2^ Ebede et al similarly noted paraffin sections’ value in evaluating high-risk NMSC tumors and unusual histologic findings on MMS frozen sections.^3^ Additionally, evidence suggests that perineural inflammation on Mohs frozen sections may indicate underlying perineural invasion, warranting permanent histologic investigation.^4^

On the other hand, a recent study examining the utility of permanent sections for MMS of SCC found that paraffin-embedded sections did not result in clinically meaningful changes in management.^5^ However, immune status was not evaluated, and the study was limited to SCC alone and did not include other NMSCs. Thus, while universal paraffin section submission in all NMSC cases may not be an appropriate use of time and resources, it is prudent in select high-risk cases. Overall, our study supports the role of permanent sections in MMS when used deliberately in high-risk NMSC, ensuring optimal management and patient care.

Nevertheless, additional prospective studies are needed to determine the proper indications and guidelines for the use of paraffin sections in Mohs surgery for NMSC.

## Conflicts of interest

None disclosed.
